# Rheological and dielectric properties of different gold nanoparticle sizes

**DOI:** 10.1186/1476-511X-10-208

**Published:** 2011-11-11

**Authors:** Mohamed Anwar K Abdelhalim, Mohsen M Mady, Magdy M Ghannam

**Affiliations:** 1 Department of Physics and Astronomy, College of Science, King Saud University, P.O. 2455, Riyadth 11451, Saudi Arabia; 2 Biophysics Department, Faculty of Science, Cairo University, 12613 Giza, Egypt

**Keywords:** Gold nanoparticles, rheological parameters, size, temperature, dielectric, conductivity

## Abstract

**Background:**

Gold nanoparticles (GNPs) have found themselves useful for diagnostic, drug delivery and biomedicine applications, but one of the important concerns is about their safety in clinical applications. Nanoparticle size has been shown to be an extremely important parameter affecting the nanoparticle uptake and cellular internalization. The rheological properties assume to be very important as it affects the pressure drop and hence the pumping power when nano-fluids are circulated in a closed loop. The rheological and dielectric properties have not been documented and identified before. The aim of the present study was to investigate the rheology and the dielectric properties of different GNPs sizes in aqueous solution.

**Methods:**

10, 20 and 50 nm GNPs (Product MKN-Au, CANADA) was used in this study. The rheological parameters were viscosity, torque, shear stress, shear rate, plastic viscosity, yield stress, consistency index, and activation energy. These rheological parameters were measured using Brookfield LVDV-III Programmable rheometer supplied with temperature bath and controlled by a computer.

**Results:**

The shear stress and shear rate of GNPs have shown a linear relationship and GNPs exhibited Newtonian behaviour. The GNPs with larger particle size (50 nm) exhibited more viscosity than those with smaller particle sizes (10 and 20 nm). Viscosity decreased with increasing the temperature for all the examined GNP sizes. The flow behaviour index (n) values were nearly ≤ 1 for all examined GNP sizes. Dielectric data indicated that the GNPs have strong dielectric dispersion in the frequency range of 20-100 kHz. The conductivity and relaxation time decreased with increasing the GNP size.

**Conclusions:**

This study indicates that the GNP size has considerable influence on the viscosity of GNPs. The strong dielectric dispersion was GNP size dependent. The decrease in relaxation time might be attributed to increase in the localized charges distribution within the medium confirmed by the conductivity data. This study suggests that further experiments are required to be done after the administration of GNPs through different routes in rats in vivo.

## Introduction

Nanotechnology is enabling technology that deals with nano-meter sized objects. A study on nanoparticle is becoming a hot point owing to their novel physical and chemical attributes in electronics [1-4], optics [1,5], electro-magnetic [6]. More interests are drawn to the particular optical characteristics of nanoparticles (NPs) such as surface plasmon resonance (SPR) [7], plasmon absorption (PA) [8], surface enhanced Raman scattering [9] and resonance Rayleigh scattering [10]. These studies are very important not only for knowing about the new optical properties but also for studying the characterization and detection methods of nanoparticles.

The GNPs have unique optical properties such as distinctive extinction bands in the visible region, due to surface plasmon oscillation of free electrons [11]. The physical origin of the light absorption by GNPs is the coherent oscillation of the conduction band electrons induced by the interacting electromagnetic field. The absorption band results when the incident photon frequency is resonant with the collective oscillation of the conduction band electrons and is known as the surface plasmon resonance (SPR). The resonance frequency of this SPR is strongly dependent upon the size, shape, dielectric properties, and local environment of the nanoparticles [12-17]. This is attributable to electric dipole-dipole interaction and coupling between plasmons of neighboring particles in the dispersion.

SPR property allows the use of GNPs for many applications in the bioscience and medical fields. The GNPs are used as immunostaining marker particles for electron microscopy, and as chromophores for immunoreactions and nucleic acid hybridization [18,19]. Their application for gene delivery into cells was reported [20-23]. In addition, GNPs have attracted much attention as photo-thermal agents in hyperthermia [24].

Owing to the unique optoelectronic properties with their controlled size and morphology, GNPs find significance in the field of bionanotechnology [24] as biomarkers [25], biosensors [26], cancer diagnostic [27] and vehicles for drug delivery [24].

The size of nanomaterials is similar to that of most biological molecules; therefore, nanomaterials can be useful for both in vivo and in vitro biomedical research and applications. Therefore, an increased attention is focused on the applications of nanoparticles in biology and medicine. Therefore, it is significant for extending the characterization methods and new applications of GNPs to further study their other optical properties.

The release of drug from semi-solid carriers is influenced by the rheological behaviour as well. The effect of certain parameters such as storage time, and temperature on the quality of GNPs as pharmaceutical products can be also investigated via rheological measurements. Rheological analysis can be employed as a sensitive tool in predicting the physical properties of the GNPs sizes.

Over the past few years, dielectric properties of different NPs have been extensively investigated to get attractive information about the localized surface plasmons, and their local dielectric environment [24-28].

The objective of the present experimental work is to explore the effects of the GNP size on the rheological properties of GNPs at a fixed temperature of 37 ^°^C and over a temperature range from 37 °C to 42 °C. In addition to investigate the electrical permittivity (ε'), conductivity (σ) and loss factor (tan δ) for 10, 20 and 50 nm GNPs in the frequency range of 20 Hz to 100 kHz at room temperature.

## Materials and methods

### Gold nanoparticles size

Different GNPs sizes were purchased (Product MKN-Au) and used in this study. The GNPs sizes were in aqueous solution of size 10 nm (Product MKN-Au-010; concentration 0.01% Au), GNPs of size 20 nm (Product MKN-Au-020; concentration 0.01% Au), and GNPs of size 50 nm (Product MKN-Au-050; concentration 0.01% Au).

### Experimental set up and rheological parameters

The experimental setup for measurement of several rheological parameters of GNPs with different particle sizes (10, 20 and 50 nm). The rheological parameters were viscosity, torque, shear stress, shear rate, plastic viscosity, yield stress, consistency index, and activation energy. These rheological parameters were measured using Brookfield LVDV-III Programmable rheometer (cone-plate viscometer; Brookfield Engineering Laboratory, Incorporation, Middleboro, USA, supplied with temperature bath controlled by a computer. The rheometer was guaranteed to be accurate within ± 1% of the full scale range of the spindle/speed combination in use reproducibility is within ± .2%.

Rheological parameters were measured at started temperature of 37°C and at a gradually increase of temperature to 42°C. Temperature inside the sample chamber was carefully monitored using a temperature sensor during the viscosity measurements.

A cone and plate sensor having a diameter of 2.4 cm with an angle of 0.8 was used. The rheometer was calibrated using the standard fluids. This viscometer has a viscosity measurement range of 1.5-30,000 mPas and can handle the viscosity measurement results within the temperature range of this experiment.

The spindle type (SC-40) and its speed combinations will produce results with high accuracy when the applied torque is in the range of 10% to 100% and accordingly the spindle is chosen.

0.5 ml of each GNP size in aqueous solution was poured in the sample chamber of the rheometer. The spindle was immersed and rotated in these gold nanofluids in the speed range from 50 to 250 RPM in steps of 20 minutes. The viscous drag of the GNP aqueous solution against the spindle was measured by the deflection of the calibrated spring.

### Plastic viscosity and yield stress

The flow curves were plotted between shear stress (dyne/cm^2^) and shear rate (s^-1^) for each gold nanoparticle size. Plastic viscosity and yield stress were calculated from the linear fitting of the flow curves [29,30].

### The flow activation energy

The effect of temperature on the gold nanofluids viscosity has been described by the Arrhenius type equation [29]:

$$\eta =\eta {\text{e}}^{\text{(Ea/RT)}}$$

Where η is the viscosity, η_0 _is the viscosity at reference temperature, E_a _is the flow activation energy, R is the universal gas constant, and T is the absolute temperature. The E_a _for the flow was determined for 10, 20 and 50 nm GNPs.

### The electrical parameters

The electrical parameters were measured in the frequency range of 20 Hz up to 1 MHz using a WAYNE KERR precision component analyzer, model 6440 B (UK). The sample cell has two squared platinum black electrodes each having an area of 1 × 1 cm^2 ^with an inner electrode distance of 1 cm. The measurements were performed at 20°C. For a dielectric material placed between two parallel plate capacitor, the measured values of capacitance (C) and resistance (R) were used to calculate the real (*ε'*) and imaginary part (*ε"*) of the complex permittivity ε*=ε'-jε", while conductivity (σ) and the relaxation time ^(^**τ**) were calculated using the following equations:

**i) **ε'=ε_0_C*k *k = lcm^-1^

Where k, is the cell constant which depends on the cell dimensions

ii) Loss tangent tan*δ*=*ε"/ε' = 1/2πfRC *so, *ε"=ε'*tan*δ*

iii) The conductivity *σ*=*k*/*R*(Ω^-1^*m*^-1^)

iv) Relaxation time *τ = *1/2*πf_c_*

*f_c _*is the critical frequency corresponding to the mid point of the dispersion curve. If any dielectric material is introduced between the two plates, the corresponding response to a sinusoidal field will be characterized by dielectric properties (dielectric permittivity ε, and conductivity σ) which vary with frequency. The charge and current densities induced in response to an applied electric field is an example of an idealized parallel plate.

## Results and discussion

### Size and morphology of different gold nanoparticles

**Figure 1:**
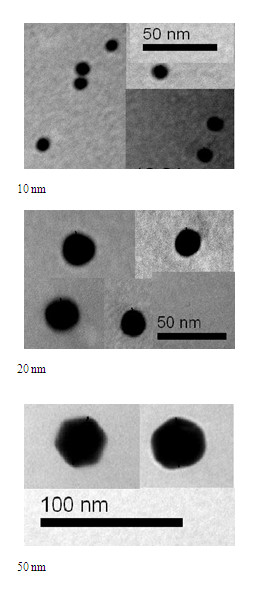
**TEM images for 10, 20 and 50 nm gold nanoparticle samples**.

The 10 and 20 nm GNPs show spherical morphology while 50 nm GNPs show hexagonal morphology, and with good particle size distribution dispersed in the solution (Figure 1).

**Figure 2:**
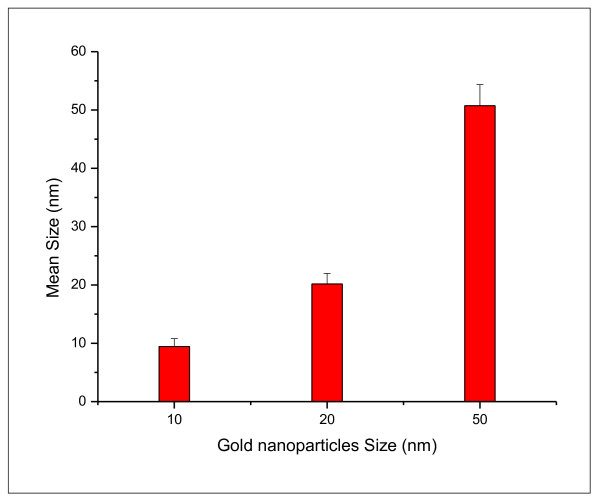
**The size of 10, 20 and 50 nm gold nanoparticles**.

The mean sizes for these GNPs were calculated from the images taken by the transmission electron microscope (TEM) for 10, 20 and 50 nm GNPs (Figure 2). Figure 2 shows that GNPs with mean size 9.45 ± 1.33 nm for GNPs size of 10 nm; 20.18 ± 1.80 nm for GNPs of size 20 nm and 50.73 ± 3.58 nm for GNPs of size 50 nm. The high electron densities of GNPs as well as the homogeneity of the particles shape and size make them highly conspicuous under the TEM. In addition, relatively simple methods can be used to obtain populations of GNPs of different average sizes, which allow simultaneous detection of several targets.

### Rheological parameters measurement

**Figure 3:**
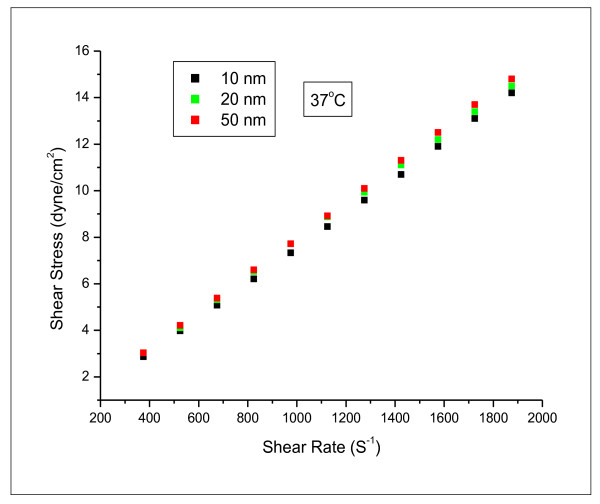
**The relation between shear rate and shear stress for 10, 20 and 50 nm gold nanoparticle sizes at temperature of 37°C**.

The rheological parameters of different GNPs sizes were measured. The relationship between shear stress and shear rate for different GNPs sizes at fixed temperature of 37 °C was measured (Figure 3). The relationship between shear stress and shear rate for different GNPs sizes was linearly related, and GNPs were exhibited Newtonian behaviour. The linear flow rate relation for the different GNPs sizes were described by the following equations: **10 nm: **Y = 0.00743 × + 0.077; **20 nm: **Y = 0.00775 × + 0.104 and **50 nm: **Y = 0.00793 × + 0.038

The plastic viscosity (a measure of the internal resistance to fluid flow of a Bingham plastic, expressed as the tangential shear stress in excess of the yield stress divided by the resulting rate of shear) [29], yield stress (dyne/cm^2^) (the minimum stress needed to cause a Bingham plastic to flow), consistency index k (an indication of the viscous nature of gold nanoparticles), the flow behaviour index (n) (a measure of departure from Newtonian flow) and activation energy (kJ/mol) were calculated for different GNPs sizes as shown in table 1.

**Table 1:** 

Gold nanoparticle size (nm)	10 nm	20 nm	50 nm
**Plastic viscosity (cP)**	0.743 ± 0.003	0.775 ± 0.003	0.793 ± 0.003
**Yield stress (dyne/cm^2^) **	0.077 ± 0.040	0.104 ± 0.030	0.038 ± 0.048
**Consistency index (cP) (k)**	0.727 ± 0.022	0.92 ± 0.029	0.846 ± 0.017
**Flow index (n)**	1.005 ± 0.0040	0.997 ± 0.0043	0.991 ± 0.0026
**Activation Energy (kJ/mol)**	332.55	415.40	182.20

These parameters were calculated from fitting the experimental data for the different GNPs sizes. n and k values were calculated from equation (1). The yield stress was increased from 0.038 ± 0.048 dyne/cm^2 ^for GNPs of size 50 nm to 0.104 ± 0.030 dyne/cm^2 ^for GNPs of size 20 nm. While the plastic viscosity decreased from 0.793 ± 0.003 for GNPs of size 50 nm to 0.775 ± 0.003 for GNPs of size 20 nm. The n and k values were ranged from 0.991 ± 0.0026 to 1.005 ± 0.0040, and from 0.846 ± 0.017 to 0.727 ± 0.022, respectively. The values of the flow behaviour index (n) were equal ≤ 1 for all the different GNPs sizes.

The rheological properties for the different GNPs sizes in aqueous solution can be described by this power law model [30]:

(1) $$\text{t = k}\gamma^{\text{n}}$$

Where τ is the shear stress, k is the consistency index, γ is the shear rate, and n is the flow behaviour index. k can be used to describe the variation in plastic viscosity for the different GNPs sizes [30].

**Figure 4:**
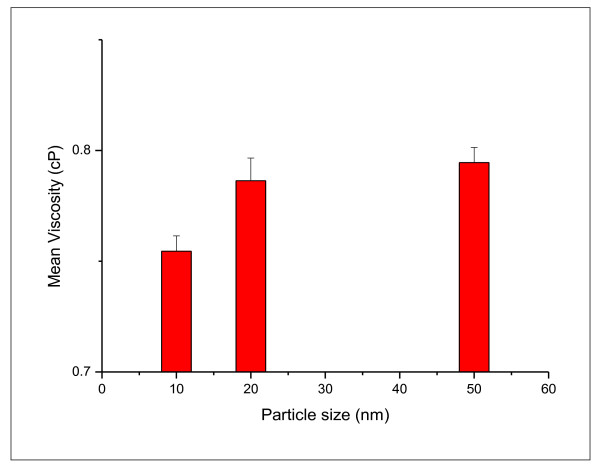
**variation of mean viscosity for 10, 20 and 50 nm gold nanoparticles**.

The mean viscosity measured for the different GNPs sizes at wide range of shear rate (375-1875 s^-1 ^is shown in Figure 4. The mean viscosity measured for different GNPs sizes increased with increasing the GNP size. Based on the present results of GNPs rheological properties, it can be concluded that increase the size of GNPs would cause an increase in the GNPs viscosity.

**Figure 5:**
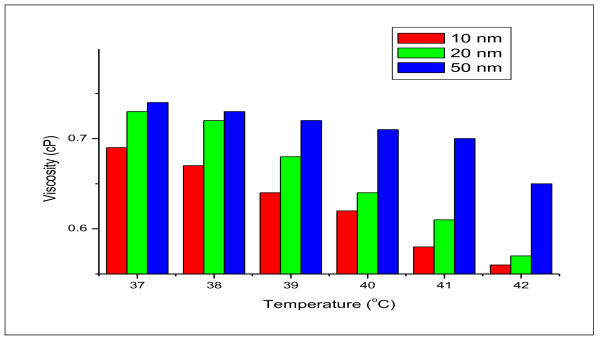
**variation of viscosity for 10, 20 and 50 nm gold nanoparticles versus the temperature**.

The measured viscosity for all GNPs sizes has shown a decrease with increase in the nanoparticles temperatures (37 °C to 42 °C), as can be observed from Figure 5. The GNPS with large size (50 nm) were exhibited higher viscosity.

**Figure 6:**
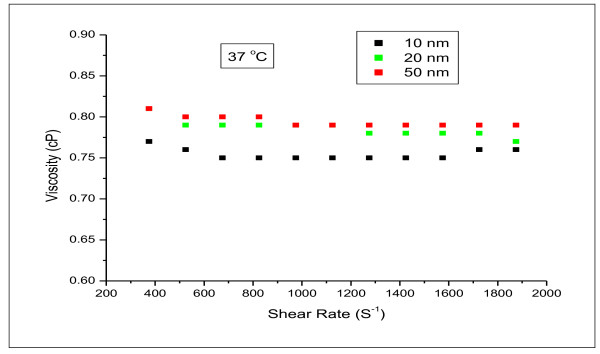
**variation of the viscosity with shear rate for 10, 20 and 50 nm gold nanoparticles**.

The viscosity of different GNPs sizes were not altered for different shear rates at fixed temperature of 37°C as shown in Figure 6.

### The dielectric Measurements

**Figure 7:**
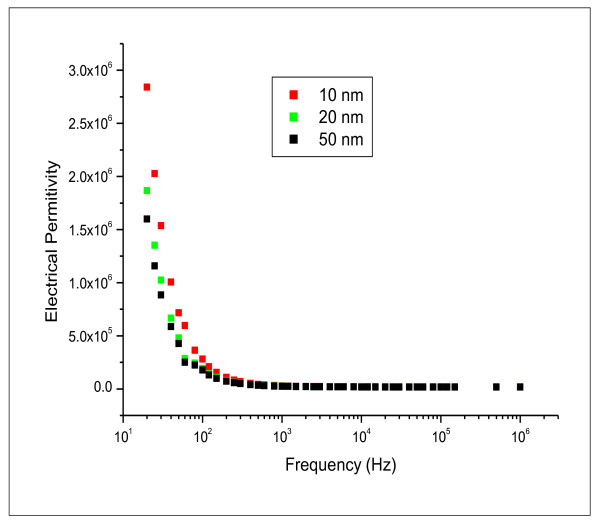
**relative permittivity ε' as function of the applied frequency in the range of 20 Hz to 1 MHz for 10, 20 and 50 nm gold nanoparticles**.

**Figure 8:**
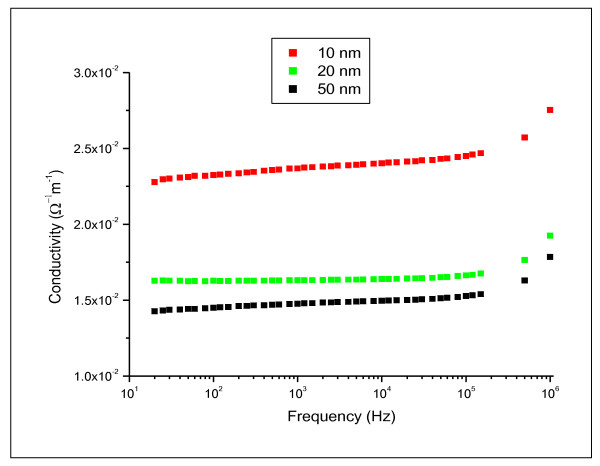
**electrical conductivity (σ) as function of the applied frequency in the range of 20 Hz to 1 MHz for 10, 20 and 50 nm gold nanoparticles**.

Figures 7 and 8 show the variation of electrical permittivity (ε') and conductivity (σ) with frequency at room temperature for different GNPs sizes 10, 20 and 50 nm. The presented dielectric data indicates that the GNPs have strong dielectric dispersion corresponding to the alpha relaxation region in the frequency range of 20 Hz to 100 kHz which identified as anomalous frequency dispersion. A rapid decrease in the dielectric constant may be attributed to the tendency of dipoles in GNPs to orient themselves in the direction of the applied field in the low-frequency range. However, in the high-frequency range the dipoles will hardly be able to orient themselves in the direction of the applied field and hence the value of the dielectric constant is nearly constant. Moreover, the conductivity decreases as the size of GNPs increases. At high frequency, the conductivity increased rapidly for all the examined GNPs size.

**Figure 9:**
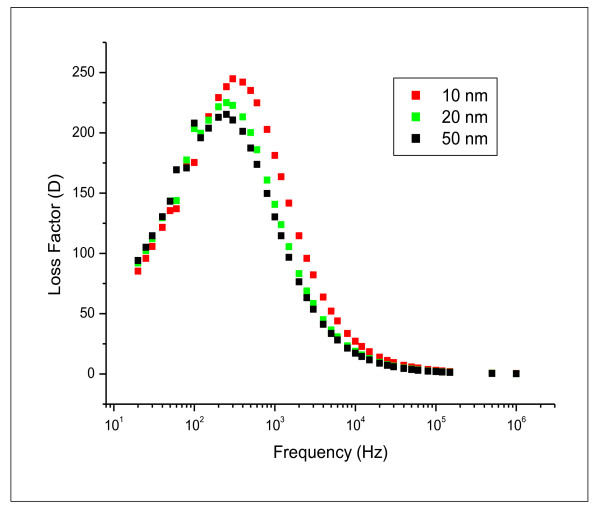
**the variation of loss factor (tanδ) with the applied frequency in the range of 20 Hz to 1 MHz for 10, 20 and 50 nm gold nanoparticles**.

The variation of loss factor (tanδ) as a function of frequency for the different GNPs sizes is shown in Figure 9. It is clear that GNPs show a relaxation process. The relaxation time was found to decrease with the increase of GNPs size and was found to be 2.5, 3.5 and 4 ms for 10, 20 and 50 nm GNP size, respectively. This may be attributed to the increase in the localized charges distribution within the medium which was confirmed from the conductivity data (Figure 9).

Further studies are required to be done after the administration of GNPs through different routes in the rats in vivo.

## Conclusions

The 10, 20 and 50 nm GNPs were used in the present study. The rheological and the dielectric properties of these GNPs were investigated.

Our findings revealed that the increase in viscosity was GNPs size dependent. The shear stress and shear rate of GNPs have shown linear relationship and exhibited Newtonian behaviour. The GNPs with larger size (50 nm) exhibited higher viscosity values than those with smaller particle sizes (10 and 20 nm).

Viscosity of GNPs decreased with increasing the temperature for all the examined GNPs sizes. The values of the flow behaviour index (n) were ≤ 1 for all examined GNPs sizes.

The dielectric data indicated that GNPs have strong dielectric dispersion in the frequency range of 20 Hz-100 kHz which was particles size dependent. Moreover, the conductivity increased with increasing the size of GNPs.

The relaxation time decreased with increasing the GNP size which may be attributed to increase in the localized charge distribution within the medium confirmed by the conductivity data.

## Competing interests

The authors declare that they have no competing interests.

## Authors' contributions

MAKA, MMM and MMG have analyzed data, interpreted and written the final draft of this manuscript. The animal model used in this study was obtained from the Laboratory Animal Center (College of Pharmacy, King Saud University, Saudi Arabia). MAKA has conceived the study and its design and obtained research grants for this study. The authors have read and approved the final manuscript.
